# The method to divide a sentence of requirement into individual requirements and the development of requirement specification editor which can describe individual requirements

**DOI:** 10.1186/2193-1801-2-145

**Published:** 2013-04-05

**Authors:** Kuniya Sato, Masahiro Ooba, Tomohiko Takagi, Zengo Furukawa, Seiichi Komiya, Rihito Yaegashi

**Affiliations:** Graduate school of Engineering, Kagawa University, Takamatsu-city, Kagawa, Japan; Faculty of Engineering, Kagawa University, Takamatsu-city, Kagawa, Japan; Engineering and Design, Shibaura Institute of Technology, Minato-ku, Tokyo, Japan

## Abstract

**Background:**

Agile software development gains requirements from the direct discussion with customers and the development staff each time, and the customers evaluate the appropriateness of the requirement. If the customers divide the complicated requirement into individual requirements, the engineer who is in charge of software development can understand it easily. This is called division of requirement. However, the customers do not understand how much and how to divide the requirements.

**Results:**

This paper proposes the method to divide a complicated requirement into individual requirements. Also, it shows the development of requirement specification editor which can describe individual requirements.

**Conclusions:**

The engineer who is in charge of software development can understand requirements easily.

## Introduction

Generally, the software is developed according to a life-cycle model (Boehm 1988; DeMarco 1979). Any life-cycle models certainly have the software requirement specification(SRS) and software test. The purpose of SRS is to gain requirements from customers (Karl 2003; IEEE Computer Society 1990), and the purpose of software test is to improve the quality (Beck 2003). The test cases used by a software test are generated based on the SRS. If the engineer who takes charge of SRS and the engineer who takes charge of software test have a different understanding from the same SRS, they may generate low-quality test cases. It will also lead to low-quality software.

Recently, MBT model-based testing (Doungsa-ard et al. 2007; Kakaji et al. 2009) has been proposed. MBT is the method to test using test case which create based on model of software behavior. A method is suggested under this situation. It describes the requirement specification of software as a model, and create a test case based on the model. In order to describe the requirement specification as a model, it is necessary to divide the various forms of requirements and make the model correctly.

Agile software development gains requirements from the direct discussion with customers and the development staff each time, and the customers evaluate the appropriateness of the requirement. If the customers divide the complicated requirement into individual requirements, the engineer who is in charge of software development can understand it easily. This is called division of requirement (Sillitti and Succi 2005). However, the customers do not understand how much and how to divide the requirements.

A part of 1.2 scope shows the requirement sentences which is written on the requirement specification. These sentences include several requirements such as “The teacher can send a mail” and “The staff can receive a mail”. However, it does not have the rule about how much the user should divide the requirement sentences. It depended on the person who made the requirement specification. If one of the requirement sentences include several requirements, the developer cannot understand the requirement of system correctly. A software which does not satisfy the customer’s requirement will be developed, and it causes the return during the software development.

The test case in software test is generally creates based on the requirement specification. If one of the requirement sentences includes several requirements, the engineer in charge of the software test cannot understand the requirement. Then, the test case is creates although the customer’s requirement is not correctly confirmed. It becomes a cause to decay the quality of software.

Recently, the software test make wide use of test management system. TestLink (Teamst 2012) is a famous test management system. TestLink is a web-based test management system that facilitates software quality assurance. It is developed and maintained by Teamst. The platform offers the support for test cases, test suits, test plans, test projects and user management, as well as various reports and statistics. TestLink has the function which relates requirement specification document to test case. Normally, a sentence of requirement specification document has some requirements. TestLink can only relate a sentence of requirement specification to a test case. TestLink needs the division of requirements to relate the customer’s requirement to the test case correctly.

This paper proposes the method to divide the requirements by establishing how to describe the division requirements. This paper describes that the sentence of SRS can be divided into six parts, 5W1H, (Actor(Who), Time(When), Location(Where), Reason(Why), Object of target(What)), and SRS has the tag which 5W1H are written as semantic information. We developed the Software requirement specification(SRS) editor system with semantic information. Since the system which we developed can have semantic information, the engineer who takes charge of SRS and the engineer who takes charge of software test have the same understanding.

## Method

### The method to divide a complicated requirement sentence into individual requirements

**Figure 1:**
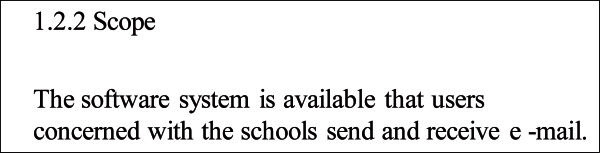
**The method to divide a complicated requirement sentence into individual requirements.**

A part of 1.2 scope shows that the people who belong to university can send and receive a mail. However, this requirement sentence does not show who these people are clearly. Figure 1 shows an example which this requirement is divided into individual requirements. If they are teachers, staffs and students, the requirement can be divided into “The teachers can send and receive a mail.”, “The staffs can send and receive a mail.” and “The students can send and receive a mail.” Also, ”Send and Receive” can be divided into “Send” and “Receive”. We can understand this sentence is divided into 6 individual requirements, “The teachers can send a mail.”, “The teachers can receive a mail.”, “The staffs can send a mail.”, “The staffs can receive a mail.”, “The students can send a mail.” and “The students can receive a mail.”

In order to divide a complicated requirement into individual requirements, this chapter suggests the method to describe the individual requirements. Sugimoto (Sugimoto et al. 2007) described that the sentence of SRS can be divided into five parts, 4W+non-functional requirement (Actor(Who), Time(When), Location(where), Reason(Why), and non-functional requirement). This paper proposes that the sentence of SRS can be divided into six parts, 5W1H, (Actor(Who), Time(When), Location(where), Reason(Why), Object of target(What)) based on Sugimoto’s proposal, and SRS has the tag which 5W1H (Media Awareness Network 2008) are written as semantic information. 5W1H are questions whose answers are considered basic in information-gathering. They are often mentioned in journalism (cf. news style), research, and police investigations. Because our method use 5W1H.

The semantic information is described by the tag of XML form. The user can define the several tags by XML, and give the meanings to these information. Also, it is easy to share the structured documents and data among different information system, especially via internet. XML enables us to write the structured documents. We can define the minimum of individual requirement.

**Figure 2:**
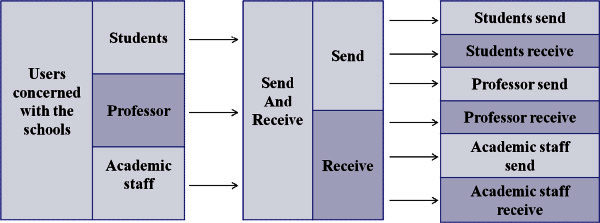
**The complicated requirement into individual requirements using XML.**

Figure 2 shows the sample which several requirements are divided into individuals. However, semantic information does not have the description about the degree of requirement. It is possible for the engineer who takes charge of SRS and the engineer who takes charge of software test to have the different understanding. This paper proposes are to divide the sentence of SRS into the six parts, to give the degree of requirement in SRS, and to have the degree of requirement as semantic information. This paper also proposes the sentence of SRS to define four degree requirement. This paper shows that the degree of requirement can be divided into four, those are Must, Shall, Will, Can. However, the system of SRS editor can set up the degree of requirement freely, for example, a number can also be used.

Subsection ‘The SRS with semantic tag in XML’ is the example which a requirement sentence is described by a tag of XML and a tag of requirement degree is added. This example shows not only the requirement that “ The student can send a mail.” but also when and where this requirement should be satisfied. It means that the requirement is the student can always receives a mail not only at school but also at any places. Also, this requirement has the tag of “must”. It means that this requirement must be realized.

### A part of 1.2 scope

#### 1.2.2 Scope

The software system is available that users concerned the schools send and receive e-mail.

##### The SRS with semantic tag in XML



### Requirement specification editor system with semantic information

**Figure 3:**
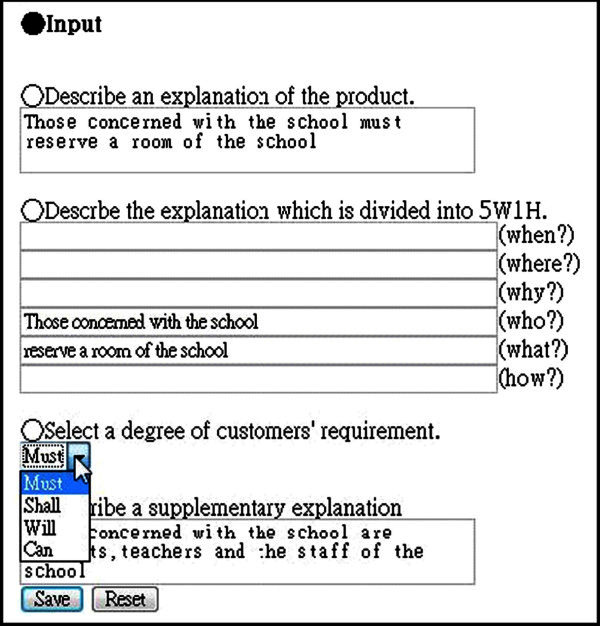
**Requirement specification editor.**

The requirement specification editor which we developed can not only make SRSs based on IEEE std 830-1998 (IEEE 1998), but also make SRSs with semantic information by use of tag. The editor is used in the situation which acquisition of the requirement from a customer finished. Figure 3 shows requirement specification editor, and subsection The SRS with semantic tag in XML shows requirement specification which requirement specification editor create. Subsection The SRS with semantic tag in XML shows that the system written in this SRSs can send e-mail, but one can not understand that user who can send(student?, professor?, staff?). The system which we developed have semantic information with tag, the system can be find out lack of description.

Requirement Specification don’t know “when user can send e-mail”,“where user can send e-mail”, “how user can send e-mail”. But the requirement specification editor which we developed not only divide a complicated requirement sentence into individual requirements, but also can know “when user can send e-mail”,“where user can send e-mail”, “how user can send e-mail”. This means that student can send e-mail “any time” and “anywhere”. And that is, Student can send e-mail not only inside campus, and also outside campus. The requirement specification editor which we developed can express detail of Requirement. And This XML have tag of <must> </must>. This tag means the degree of requirement. <must> </must> means the requirement which must be attained. Requirement specification can not express degree of requirement. Software test check satisfaction of customer requirement. Generally, customer have many kind of requirement. for example, requirement which customer must satisfy, and so on. The requirement specification editor which we developed can express degree of requirement.

### The function which reuses the inputted the description and the function which shows the relationship of the descriptions

This section describes the function which reuses the inputted the description and the function which shows the relationship of the descriptions. There are many problems such someone who operate are not indicated and a different description is used in the same meaning, and expression is ambiguous in SRSs. The Editor which we developed have the function which reuses the inputted the description. This function has an effect which prevents using description which is different in the same meaning.

**Figure 4:**
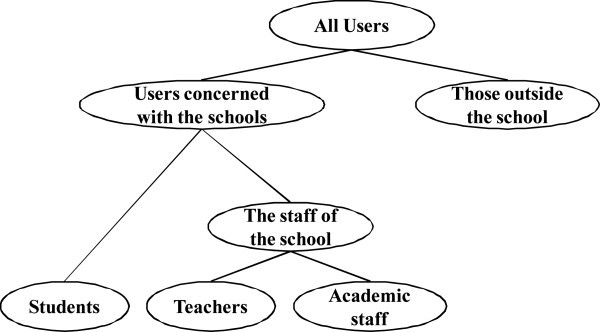
**The function which shows the relationship of the descriptions.**

Change of SRS sometimes takes place. However, it is difficult to know the influence by change of a requirement. The system which we developed have the function which shows the relationship of the descriptions. Figure 4 shows the function which shows the relationship of the descriptions. The staff of a school is education staff and administrative staff. If administrative staff changes, the staff of a school may also change and the people involved in the school may also change. In this way, by describing the association between the word, you can find a lack of information.

## Conclusion

This paper proposed the method to divide a complicated requirement into individual requirements. Also, it presented the development of requirement specification editor which can describe individual requirements.

This paper described that the sentence of Software requirement specification(SRS) can be divided into six parts, 5W1H, (Actor(Who), Time(When), Location(where), Reason(Why), Object of target(What) and requirement specificaton have tag which 5W1H was written as semantic information. This paper also describes how to given the degree of requirement, and describes the methods to have tag which the degree of requirement was written as semantic information. This paper describes the Requirement Specification editor system which we developed with semantic information, the function which reuses the inputted the description, the function which shows the relationship of the descriptions.
